# Modeling of Noise and Resistance of Semimetal Hg_1-x_Cd_x_Te Quantum Well used as a Channel for THz Hot-Electron Bolometer

**DOI:** 10.1186/s11671-016-1405-x

**Published:** 2016-04-11

**Authors:** E. O. Melezhik, J. V. Gumenjuk-Sichevska, F. F. Sizov

**Affiliations:** Department 38, Institute of Semiconductor Physics of NAS of Ukraine, 03028, pr. Nauki 41, Kyiv, Ukraine

**Keywords:** THz, Bolometers, NEP, HgCdTe, Noise, 73, 73.63.Hs, 73.50.Td

## Abstract

Noise characteristics and resistance of semimetal-type mercury-cadmium-telluride quantum wells (QWs) at the liquid nitrogen temperature are studied numerically, and their dependence on the QW parameters and on the electron concentration is established. The QW band structure calculations are based on the full 8-band *k.p* Hamiltonian. The electron mobility is simulated by the direct iterative solution of the Boltzmann transport equation, which allows us to include correctly all the principal scattering mechanisms, elastic as well as inelastic.

We find that the generation-recombination noise is strongly suppressed due to the very fast recombination processes in semimetal QWs. Hence, the thermal noise should be considered as a main THz sensitivity-limiting mechanism in those structures. Optimization of a semimetal Hg_1-x_Cd_x_Te QW to make it an efficient THz bolometer channel should include the increase of electron concentration in the well and tuning the molar composition *x* close to the gapless regime.

## Background

The problem of creation of fast and sensitive detectors of THz spectral range is important for various areas such as medicine, security, and aerospace. Among other un-cooled or moderately cooled detector types, semiconductor bolometric detectors allow one to combine high sensitivity, operation speed, and devise compactness and durability.

Mercury-cadmium-telluride (MCT) quantum wells (QWs) are promising materials for the implementation of the channel for thermal direct detectors, because Hg_1-x_Cd_x_Te QWs can be characterized by high electron mobility and concentrations even at liquid nitrogen temperatures [1]. Detectors based on a quantum well (QW) are a good choice, because the momentum quantization in the QW growth direction allows for a significant reduction of the 2D electron heat capacity. The requirement of a high sensitivity and low noise could be obtained by using a low-resistive channel. High operation speed could be realized for high-mobility channels, or for channels with a fast energy relaxation of the 2D electron gas (2DEG).

Hg_1-x_Cd_x_Te QWs can realize semimetallic or semiconducting states, depending on their molar composition x and QW width *L* [2]. Semimetallic state is characterized by much higher conduction electron concentration at the liquid nitrogen temperature [3]. Therefore, semimetallic QWs can have much lower resistivity and lower noise comparing with undoped semiconducting Hg_1-x_Cd_x_Te QWs of the same thickness. So we restrain our simulation for the case of semimetallic Hg_1-x_Cd_x_Te quantum wells.

To create the efficient and sensitive THz bolometers, one needs to be able to predict expected characteristics of Hg_1-x_Cd_x_Te quantum well channel. To our knowledge, a systematic theoretical study of transport properties of semimetallic Hg_1-x_Cd_x_Te QWs at liquid nitrogen temperature as well as experimental is still lacking. Electron mobility, energy spectra, and intrinsic carrier concentrations in the n-type Hg_0.32_Cd_0.68_Te/Hg_1-x_Cd_x_Te/Hg_0.32_Cd_0.68_Te (QW) in semimetallic state we have modeled numerically in [1]. Our modeling has shown that high electron mobility can be obtained at high electron concentration in the well, which enhances 2D electron gas screening and decreases hole concentration.

Based on these results, we aim at finding the best parameters of Hg_1-x_Cd_x_Te QW as a channel of hot-electron bolometer for THz detection. For this purpose, we estimate transport and noise properties of such channels in the dependence of QW properties in the present paper. We model the resistance and noises in Hg_0.32_Cd_0.68_Te/Hg_1-x_Cd_x_Te/Hg_0.32_Cd_0.68_Te QW in the dependence on the well chemical composition x, thickness, and electron concentration at liquid nitrogen temperature (*T* = 77 K). Also we estimate noise equivalent power (NEP) of bolometric detectors utilizing considered structures as a channel, for the frequency of incident radiation of 140 GHz.

These estimates are useful not only for thermal-type detector such as bolometer but also for rectifying one as field-effect transistor with high electron mobility (HEMT), where an increase of the electron concentration could be achieved by applying the gate bias voltage.

In this work, we also compare estimated characteristics of semimetal-type HgCdTe THz hot-electron bolometer with semiconductor-type MCT and with graphene HEBs.

## Methods

Simulations of the energy spectra and wave-functions were performed in the framework of the 8-band *k.p* Hamiltonian [4] to incorporate strong band mixing and nonparabolicity of carrier dispersion law. Such modeling allows one to describe the presence of semimetallic or semiconducting states in the well. In our modeling [1, 3], we consider Hg_1-x_Cd_x_Te quantum wells with different *x*, grown in (001) plane, where lattice mismatch strains are compensated. Barrier layers have composition *x* = 0.68 (Hg_0.32_Cd_0.68_Te). Level of background charged impurities in QW is taken to be stable, it equals to 10^15^ cm^−3^. We have modeled numerically the energy spectra and intrinsic carrier concentrations in the n-type Hg_0.32_Cd_0.68_Te/Hg_1-x_Cd_x_Te/Hg_0.32_Cd_0.68_Te quantum well (QW) in semimetallic state.

The three most important electron scattering mechanisms—longitudinal optical phonon scattering (inelastic), residual charged impurities scattering, and electron-hole scattering (both are elastic)—take place in bulk Hg_1-x_Cd_x_Te at nitrogen temperature [5]. To calculate the impact of these scattering mechanisms on the electron mobility in QW, the linearized Boltzmann transport equation (lBTE) was iteratively solved [1]. Direct solution of lBTE allows one to account accurately inelasticity of electron scattering and recovers how carrier distribution function is perturbed by the applied electric field in the channel. Estimation of the perturbed distribution function allows one to calculate electron mobility. We have also estimated the contributions from other scattering mechanisms involving acoustic phonons, interface roughness, alloy disorder, fluctuations of composition and effective mass, which have been found to be negligible for QW widths larger than 12 nm.

Comparing the separate impacts of each scattering mechanism, one can see that the longitudinal optical phonon scattering is strongly suppressed because of the strong dynamical screening. For an intrinsic 20-nm-wide quantum well with the composition *x* = 0, the electron mobility for the LO phonon scattering is about 3.8 * 10^6^ cm^2^/(Vs), while for n-doped quantum well of the same geometry with composition *x* = 0.06 (the electron concentration 1.5 * 10^17^ cm^−3^), the electron mobility limited by the LO phonon scattering is about 6.8 * 10^6^ cm^2^/(Vs). As these mobilities are much higher than the corresponding total mobilities, we can conclude that the main contribution to the total mobility comes from the charged impurity scattering and electron-hole scattering. Relative importance of these two scattering mechanisms can be established from the comparison of hole and charged impurity concentrations.

Our modeling [1] has shown that at the liquid nitrogen temperature, high electron mobility can be obtained at high electron concentration in the well, which enhances 2DEG screening and decreases hole concentration. Such an increase of the electron concentration could be achieved by delta-doping of barriers or by applying the top-gate bias voltage. Growth of the mobility with the increase of the quantum well composition *x* could be explained by a lower concentration of heavy holes at the same value of the electron concentration. Since the concentration of holes in QW is often higher than 10^15^ cm^−3^, the fabrication of high purity samples with low concentration of residual charged impurities (of the order of 10^14^ cm^−3^) will not improve the electron mobility sufficiently. Our modeling shows that because of the high hole concentration, the purity of samples in many configurations is of a lower importance for obtaining high electron mobility than the electron concentration in the well. This conclusion could be important for the reduction of fabrication costs for high-mobility HgCdTe heterostructures.

Our results show that the increase of the electron concentration in the well enhances the screening of the 2D electron gas, decreases the hole concentration, and can ultimately lead to a high electron mobility at liquid nitrogen temperatures. The highest mobility values (up to 10^6^ cm^2^/(Vs)) can be achieved in the Hg_1-x_Cd_x_Te at *x* = 0.09, notable near the inversion point, at high electron concentration in the well. The increase of the electron concentration in the QW could be achieved in situ by delta-doping of barriers or by applying the top-gate potential. Our modeling has shown that for low molar composition *x*, the concentration of holes in the well is high in a wide range of electron concentrations; in this case, the purity of samples does not significantly influence the electron mobility.

If to consider HgCdTe QW as a channel of hot-electron bolometer, three main noise generation mechanisms present in this case: thermal (Johnson’s) noise, generation-recombination noise, and photon noise [6]. Total noise *U*_*N*_ can be found as a mean square of these three noises. In further calculations, we use the bandwidth of the central frequency *∆f* = 1 Hz. The value of thermal noise can be found as [6]:

1 $$ \left\langle {U_J}^2\right\rangle =4R{k}_BT\varDelta f, $$

where *R* is the detector resistance, *k*_*B*_ is the Boltzmann constant, and *T* is the detector temperature.

Generation-recombination noise can be found from [6]. For the case of (001) oriented unstrained QWs which are considered in this work, the effective mass of holes is more than an order of magnitude greater than the effective mass of electrons at the Fermi level. Thus we can simplify the equation for this noise:

2 $$ \left\langle {U_{g-r}}^2\right\rangle =\frac{4{I}_c^2{p}_0\tau }{n_0\left({n}_0+{p}_0\right)\left(1+{\omega}^2{\tau}^2\right)}{R}^2\varDelta f $$

Here *τ* is the dominant lifetime, *I*_*c*_ is the current, *n*_0_ and *p*_0_ refer to the total numbers of electrons and holes in the channel at thermal equilibrium, *ω* is the circular frequency which is related to the frequency of incident radiation *f* as *ω* = 2*πf*. Photon noise was calculated with the usual framework (see [6]):

3 $$ \begin{array}{l}{U}_{ph}={\left(2A\eta N(T)\right)}^{1/2}eR,\hfill \\ {}N(T)={\displaystyle \underset{\lambda_1}{\overset{\lambda_2}{\int }}}\frac{2\pi c}{\lambda^4\left(\mathrm{E}\mathrm{x}\mathrm{p}\left[hc/\lambda {k}_B{T}_b\right]-1\right)}d\lambda, \hfill \end{array} $$

where *N* is the photon flux from the *T*_*b*_ = 300 K background hemisphere, detector radiation coupling *η* was taken to be 0.5, spectral range of radiation wavelength (*λ*_*1*_ *≤ λ ≤ λ*_*2*_) which fell on the bolometer was taken to be ±15 % from central frequency of incident radiation from the source, *A* is the antenna area, *e* is electron charge, and *c* is the speed of light.

NEP is determined as [7]:

4 $$ \mathrm{N}\mathrm{E}\mathrm{P}=\frac{U_N}{S_V{\left(\varDelta f\right)}^{1/2}} $$

where *Δf* = 1 Hz is the pass bandwidth.

## Results and Discussion

We have applied our recent calculations of electron mobility in the semimetal Hg_1-x_Cd_x_Te QW [1] to model the resistivity and noises in such QW when it is used as a channel of the THz-range hot-electron bolometer.

**Fig. 1:**
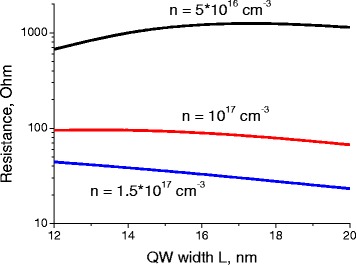
Dependence of the bolometer channel resistance on the QW thickness. Dependence of the bolometer channel resistance on the QW thickness *L* with the composition *x* = 0.06 at *T* = 77 K, for different electron concentrations *n*

**Fig. 2:**
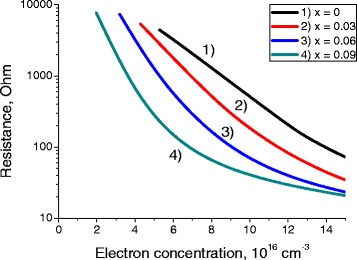
Dependence of the bolometer channel resistance on the electron concentration. Dependence of the bolometer channel resistance on the electron concentration in Hg_0.32_Cd_0.68_Te/Hg_1-x_Cd_x_Te/Hg_0.32_Cd_0.68_Te QW with the thickness *L* = 20 nm at *T* = 77 K, for different molar compositions *x*

In this case, the channel thickness corresponds to QW width *L*. Using the lateral dimensions of the bolometer channel (width *D*_w_ and length *D*_*l*_), one can calculate the channel resistivity *ρ* in a usual way as *ρ* = 1/(*eμn*), where *n* is the electron concentration, *e* is the electron charge, and *μ* is the electron mobility. Then its resistance is given by *R* = *ρD*_*l*_/(*D*_*w*_*L*). Figures 1 and 2 present the result of such calculations for a channel with *D*_w_ = *D*_l_ =50 μm.

From Figs. 1 and 2, one can see that the main impact on the resistance of the sample is made by electron concentration (Fig. 2). Variation of well thickness changes channel resistance much less significantly.

At Fig. 2, leftmost points of each curve correspond to the intrinsic case and very high resistance of samples.

Increase of electron concentration in the channel leads to substantial decrease of its resistance. It is important to outline the physical reasons of such resistance behavior to provide the technologists with the most efficient recipes of growth of low-resistance MCT heterostructures. High resistance (and low mobility) in the left-hand side of Fig. 2 is explained by high hole concentration values in an intrinsic case [1]. As electron-hole scattering is one of the most important scattering mechanisms, high hole concentration sufficiently deteriorates electron mobility and increases sample resistance.

Growth of electron concentration (at the stable concentration of background charged impurities in the well) leads to strong increase of mobility and appropriate reduction of resistance due to two simultaneous processes: decrease of hole concentration and growth of screening [1]. The channel resistance varies by more than two orders of magnitude depending on the electron concentration. Such a strong dependence could provide high sensitivity of the hot-electron bolometer, as small variations in the gate voltage should result in strong changes of the bolometer resistance. A high dynamical tenability is additional merit of the considered system as a THz detector.

When choosing QW thickness to obtain the optimal work characteristics of semimetal MCT QW channel, it could be important to avoid too thin wells (about 10 nm and less), because in that case the channel resistance and noises could be increased by interface scattering [8]. Also one should be careful with QWs in the band inversion point, as in that case additional scattering mechanism (scattering on effective mass fluctuations) could suppress the mobility and increase resistance and noises.

Low-resistance Hg_1-x_Cd_x_Te quantum wells could be obtained by n-type doping of barriers or by application of the top-gate bias to the channel.

As noises are the sensitivity-limiting mechanism, the modeling of the dependence of such noises on QW parameters is important.

There is a very few experimental data regarding the electron lifetime in semimetal HgCdTe structures. However, this time could be roughly estimated from [9–11] as 10^−10^ s.

For the noise and NEP estimates for incident radiation frequency 140 GHz, current *I*_*c*_ was taken to be 0.4 mA, in analogy to the experimental work of authors [12]. The area of antenna *A* for this frequency was estimated as $A=\frac{\lambda^2}{\left(4\pi \right)}=3.7*{10}^{-3}{\mathrm{cm}}^2$.

Using the numerical values of total noise, we could roughly estimate noise equivalent power (NEP) of the hot-electron bolometer with semimetal QW, using the experimental data for sensitivity *S*_*V*_ for semiconducting channel from [12]. For the frequency of incident radiation of 140 GHz, value of sensitivity was about 20 V/W [12].

**Fig. 3:**
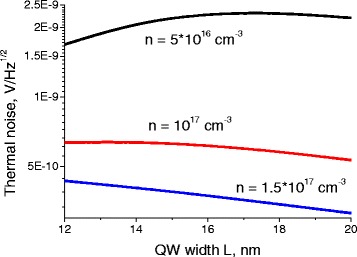
Dependence of the thermal noise on the well width. Dependence of the thermal noise in the MCT QW with molar composition *x* = 0.06 on the well width, for different electron concentrations at *T* = 77 K

**Fig. 4:**
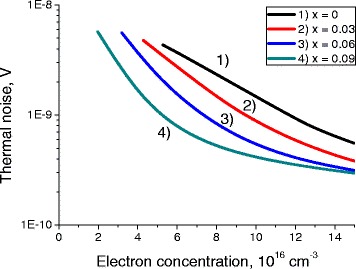
Dependence of the thermal noise on electron concentration. Dependence of the thermal noise in the QW of *L* = 20 nm width on electron concentration, for different compositions *x* at *T* = 77 K

**Fig. 5:**
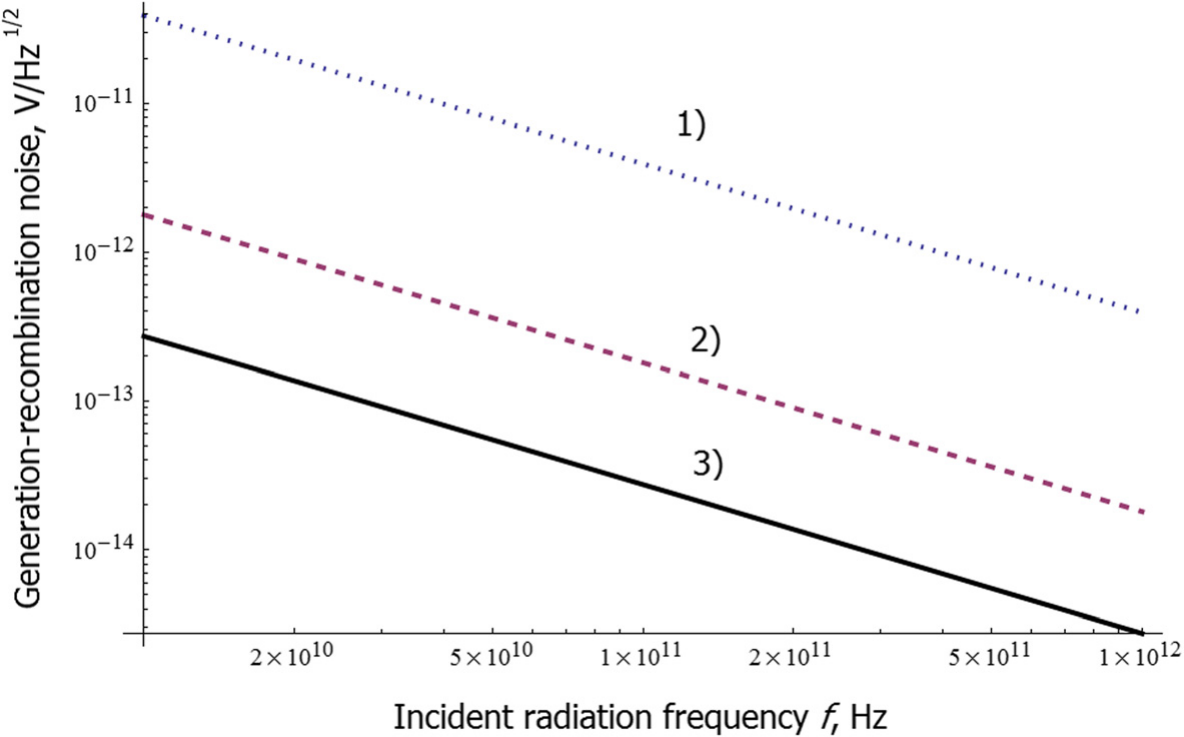
Dependence of the generation-recombination noise on incident radiation frequency. Dependence of the generation-recombination noise in QW of 20 nm width and molar composition *x* = 0.06 on incident radiation frequency, *f. I*_*c*_ = 0.4 mA. Calculations are made for three electron concentrations: (1) 7 * 10^16^ (*dotted line*), (2) 1.1 * 10^17^ (*dashed line*), and (3) 1.5 * 10^17^ cm^−3^ (*solid line*)

**Fig. 6:**
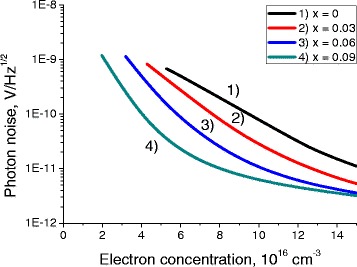
Dependence of the photon noise on the electron concentration. Dependence of the photon noise on the electron concentration in QW, for different compositions *x*. QW width is 20 nm, *T* = 77 K. Photon noise calculated in the spectral range *f* = 140 GHz ±15 %

Thus for semimetal n-type MCT QW with 20 nm width, composition *x* = 0.06 or 0.09 and electron concentration *n* = 1.5 * 10^17^ cm^−3^, using the data of Figs. 3, 4, 5, and 6 and (4), for the incident radiation frequency of 140 GHz, NEP can be estimated to be about 1.5 * 10^−11^ W/Hz^1/2^.

It is important to compare semimetal-type MCT QWs, used as a THz bolometer channel, with their direct competitors.

Comparing these semimetal-type heterostructures with semiconductor MCT layers [6], one could note that NEP is an order of magnitude lower for semimetal structures. Also, semiconductor MCT structures are characterized by much greater recombination times, about 10^−7^ s [12, 13]. Thus compared to semiconductor MCT structures, semimetal QW could provide higher bolometer operation speed (which estimates from a lifetime) and higher sensitivity.

Comparing semimetal MCT QWs with graphene, which is also often considered as a candidacy for the channel of THz bolometer, we can outline several important benefits of structures we are considering in this article. First, graphene sheets are usually characterized by resistances about 10 kOhm. This results in very strong noises, which are at least one order of magnitude stronger than noises which could be reached in optimized semimetal MCT QWs (please see Figs. 1 and 2).

Second, the high resistance of graphene layers results in quite inefficient coupling of bolometer channel with a planar metal antenna, and so leads to low sensitivity and high NEP.

Third, carrier energy relaxation process in graphene is very slow, as the only inelastic scattering mechanism present there is optical phonon scattering, and in contrast to MCT, the energy of optical phonons in graphene is more than an order of magnitude (200 meV) greater than mean thermal energy of electrons at *T* = 77 K. This results to a decrease in the efficiency of the 2DEG energy relaxation, which could deteriorate the detector operation speed and degrade the overall performance of the detector. In contrast to graphene, 2DEG energy relaxation in semimetallic Hg_1-x_Cd_x_Te QW is much faster due to low energy of the LO phonon (17 meV in HgTe), since this energy and mean electron energy are of one order. Fast 2DEG energy relaxation could be important for increasing the detector operation speed.

Fourth, graphene sheets are more sensitive to the substrate or top-gate presence. While high values (10^6^ cm^2^/(Vs)) of the mobility are measured at room temperature in exfoliated graphene sheets [14], substrate presence decreases the room-temperature mobility to measurable values of about (1…2.3) * 10^4^ cm^2^/(Vs). This also results in hard carrier density operation in graphene. In this connection, MCT-based devices can provide an order of magnitude higher mobility than graphene and much easier carrier density conduction.

Thus, semimetal HgCdTe QWs used as a channel for THz hot-electron bolometer at *T* = 77 K could provide high operation speed combined with high sensitivity and low noise.

## Conclusions

We have studied the resistance end noise dependencies of a hot-electron bolometer channel, based on *n*-type semimetallic Hg_0.32_Cd_0.68_Te/Hg_1-x_Cd_x_Te/Hg_0.32_Cd_0.68_Te quantum well, on the electron concentration and QW parameters. The channel resistance was obtained from our modeling of the well mobility. It was shown that the HEB channel resistance varies by more than two orders of magnitude (from several tens of Ohm to about 10 kOhm) depending on the QW electron concentration and by about one order of magnitude depending on the QW molar composition (in the inverted band structure range of *x*). Such a strong dependence could provide high volt-watt sensitivity of the hot-electron bolometer, as small variations in the gate voltage should result in strong changes of the bolometer resistance. A high dynamical tunability brings another benefit of the considered system for the THz detection.

We show that the generation-recombination noise in semimetal MCT quantum wells is strongly suppressed, compared to semiconducting HgCdTe samples [6]. This is caused by extremely small carrier lifetime in inverted band structure which is realized in semimetal case.

Thermal noise is the main source of the noise in these structures. Photon noise and generation-recombination noises are usually significantly smaller.

All three noises exhibit strong dependence on the electron concentration. Their level goes down with the increase of the chemical composition parameter *x* of the QW.

To obtain the optimal operation characteristics of a semimetal MCT QW channel for THz detectors, one should provide a high electron concentration in the QW, and adjust the channel chemical composition *x* to be close to the band structure inversion point (just below the inversion point, to avoid activating an additional mechanism of scattering on the effective mass fluctuations).

Our estimates for characteristics of semimetal-type HgCdTe THz hot-electron bolometers show their advantages compared to semiconductor-type MCT and to graphene HEBs. We conclude that HgCdTe semimetallic QWs can demonstrate lower resistance, lower noise values, and higher operational speed and can provide much more efficient coupling to planar antennas and much higher tunability in THz-range detector applications.

## Abbreviations

2DEG: two-dimensional electron gas
HEB: hot-electron bolometer
HEMT: high electron mobility transistor
lBTE: linearized Boltzmann transport equation
LO phonon: longitudinal optical phonon
MCT, HgCdTe: mercury-cadmium-telluride
NEP: noise equivalent power
QW: quantum well
THz: terahertz
